# Identification of a Dissection Site in the Internal Thoracic Artery Using Fluorescence Imaging: A Case Report

**DOI:** 10.7759/cureus.55199

**Published:** 2024-02-29

**Authors:** Tomohiro Nakajima, Yutaka Iba, Tsuyoshi Shibata, Akihito Ohkawa, Nobuyoshi Kawaharada

**Affiliations:** 1 Cardiovascular Surgery, Sapporo Medical University, Sapporo, JPN

**Keywords:** pci, fluorescence image, dissection, ita, cabg

## Abstract

A 66-year-old man with a history of type 2 diabetes mellitus who was undergoing hemodialysis presented with angina. Coronary angiography revealed triple-vessel coronary artery disease. He underwent multiple percutaneous coronary interventions due to recurrent restenosis and was referred for coronary artery bypass grafting (CABG). The left internal thoracic artery and bilateral saphenous veins were harvested under general anesthesia. Four CABGs were performed: left internal thoracic artery to the left anterior descending artery; saphenous vein graft to the obtuse marginal branch of the circumflex artery; and saphenous vein graft to two sites in the right coronary artery. Intraoperative assessment with transit-time flow measurements showed no abnormalities, and the surgery was completed. On postoperative day seven, coronary and graft angiography revealed dissection of the left internal thoracic artery at its midportion with restricted flow. On postoperative day eight, a surgical intervention was performed to excise the dissected segment of the left internal thoracic artery. The dissection site was identified by fluorescence imaging. The dissected segment was excised, and the artery was re-anastomosed. The postoperative course was uneventful, and graft angiography performed on postoperative day 22 confirmed good blood flow. Fluorescence imaging was valuable in identifying the dissection site in the left internal thoracic artery.

## Introduction

The early and long-term outcomes of coronary artery bypass grafting (CABG) significantly depend on graft patency [1]. Various factors contribute to graft occlusion, including graft quality, anastomotic issues, and the vascular bed distal to the anastomosis. Therefore, in CABG, grafting high-quality conduits to the appropriate target coronary arteries and evaluating the graft flow intraoperatively is crucial [2].

Transit-time flow measurement (TTFM) and intraoperative fluorescence imaging (IFI) are the most commonly used intraoperative graft assessment methods in routine clinical practice [3]. Although TTFM allows the quantitative evaluation of graft flow, it has the drawback of being influenced by arterial pressure and graft vessel diameter. On the other hand, IFI enables visual assessment of graft flow but may pose challenges for quantitative evaluation [4].

To improve graft patency rates in CABG, using the advantages of both methods to appropriately evaluate the intraoperative graft flow is essential. Prompt re-anastomosis should be performed if any issues with blood flow are identified during surgery.

## Case presentation

**Figure 1:**
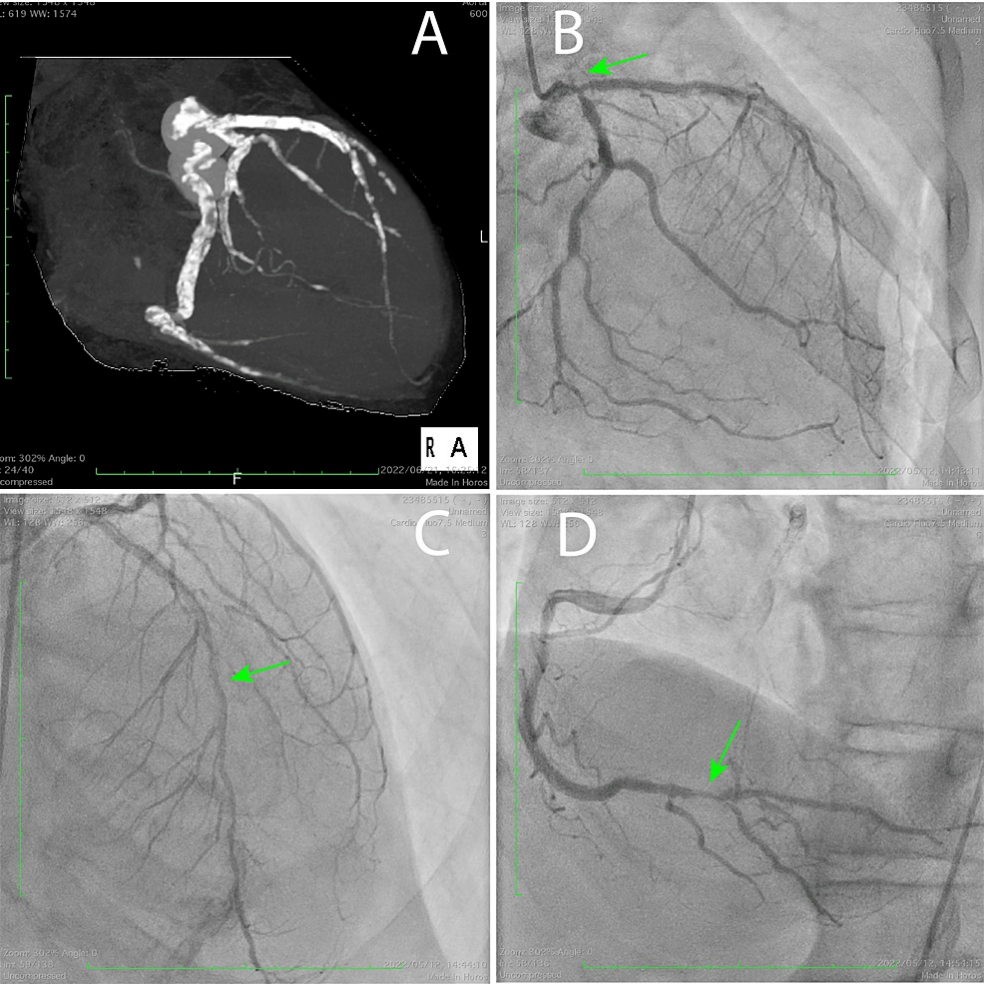
Preoperative findings. (A) Preoperative computed tomography scan maximum intensity projection. The patient exhibited triple-vessel coronary artery disease with severe calcification. (B) Left coronary artery angiography. The left main trunk had severe stenosis (green arrow). (C) Left coronary artery angiography. The left descending artery had severe stenosis (green arrow). (D) Right coronary artery angiography. Intermediate part had severe stenosis (green arrow).

A 66-year-old man with a history of type 2 diabetes and undergoing hemodialysis had a history of angina and had undergone several percutaneous coronary interventions (PCIs) in the past. Coronary angiography revealed a triple-vessel disease. Due to recurrent restenosis, he was referred to the Cardiothoracic Surgery Department for CABG. The preoperative findings are shown in Figure 1.

**Figure 2:**
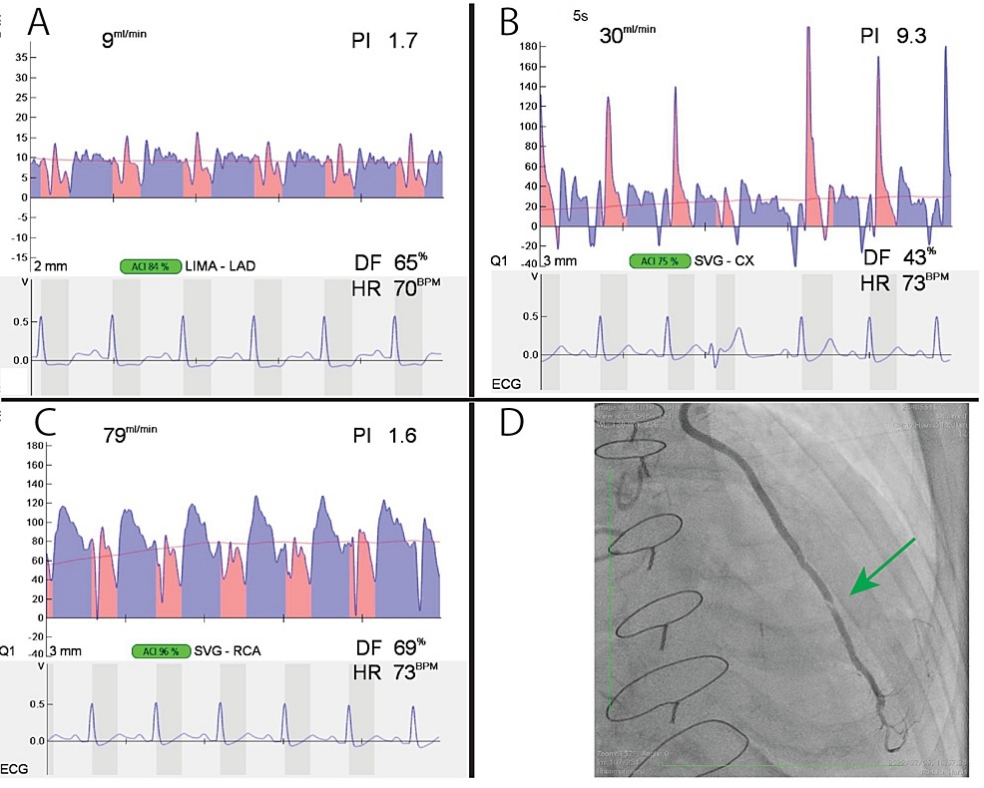
Operative findings. (A) Transit-time flow measurement of LITA-LAD was an acceptable parameter. (B) Transit-time flow measurement of SVG-LCX was an almost acceptable parameter. (C) Transit-time flow measurement of SVG-RCA was an acceptable parameter. (D) LITA-LAD angiography. The mid part of the LITA was dissected and flowed slowly (green arrow).

The coronary arteries showed significant calcification. Under general anesthesia, the decision was made to perform beating-heart coronary artery bypass surgery. Initially, the left internal thoracic artery and great saphenous veins of both lower limbs were harvested. Subsequently, the heart was arrested, and four CABGs were performed (left internal thoracic artery to the left anterior descending artery, ascending aorta to the great saphenous vein to the obtuse marginal artery, and ascending aorta to the great saphenous vein to two locations of the right coronary artery). Intraoperative assessment using TTFM showed no abnormalities in various parameters, and the surgery was completed. The results are shown in Figure 2. On the 7th postoperative day, coronary and graft angiographies were performed, which revealed dissection of the left internal thoracic artery in its midportion, resulting in restricted flow.

**Figure 3:**
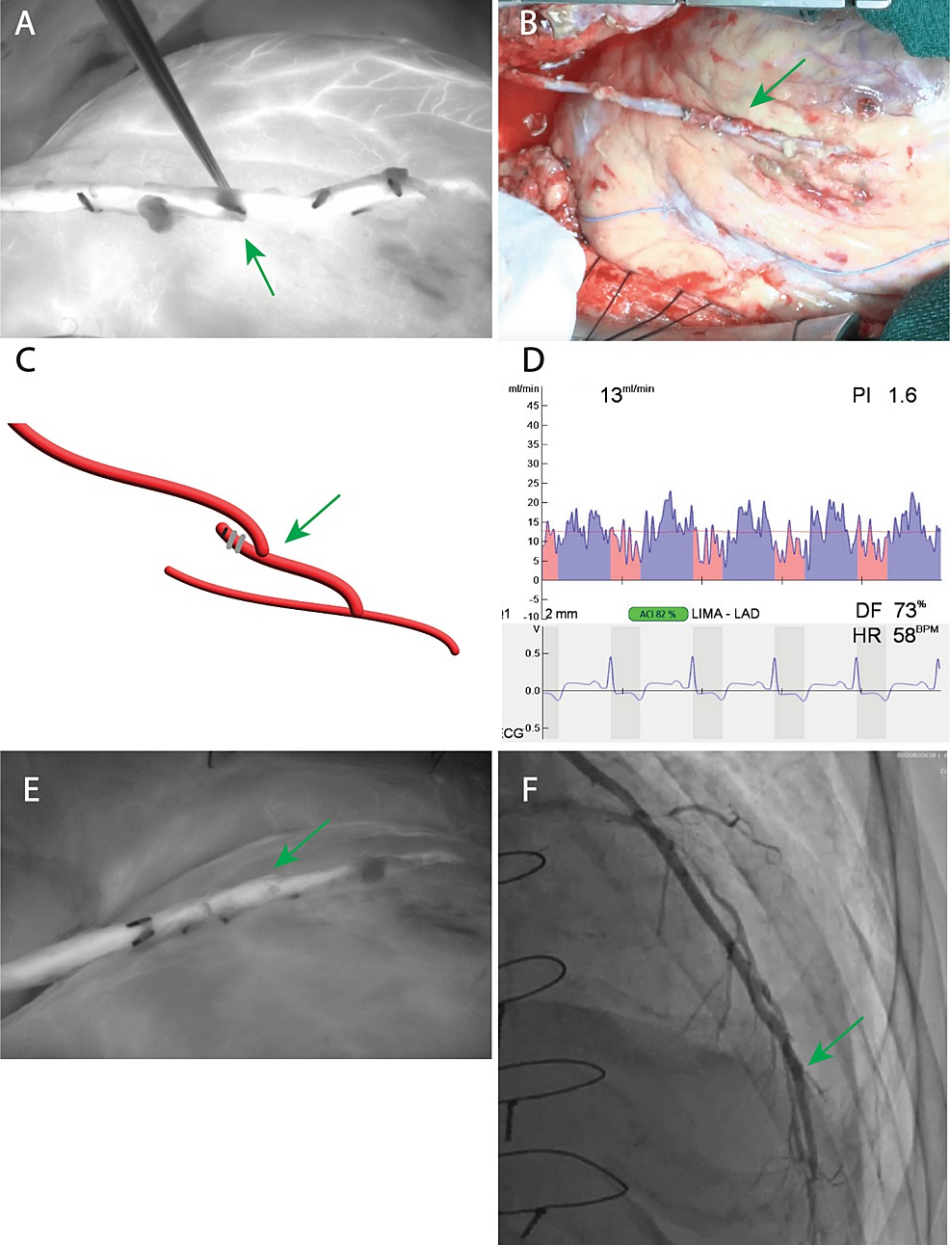
Second-operative findings. (A) Intraoperative fluorescence imaging (IFI) of LITA. The disassociation sites were immediately identified (green arrow). (B) The left internal thoracic artery was re-anastomosed by cutting off the dissection part (green arrow). (C) The operational schema revealed the anastomosis site of LAD (green arrow). (D) Transit-time flow measurement of LITA-LAD was an acceptable parameter. (E) IFI of LITA after re-anastomosis revealed good flow (green arrow). (F) LITA-LAD angiography had good flow. The anastomosed site is shown (green arrow).

To alleviate flow restriction in the left internal thoracic artery, dissection site excision surgery was performed on the 8th postoperative day. The dissection site was identified by fluorescence imaging of the left internal thoracic artery. Subsequently, the dissected segment was excised and re-anastomosed. The postoperative course was uneventful, and satisfactory blood flow was confirmed on graft angiography. Surgical and postoperative graft angiography images are shown in Figure 3.

The patient was discharged on the 22nd postoperative day. Fluorescence imaging was useful in identifying the site of the left internal thoracic artery dissection in this case.

## Discussion

CABG is a treatment option for coronary artery disease (CAD) caused by the narrowing or blockage of the coronary arteries. Typically performed by cardiac surgeons, CABG involves the creation of new blood vessels (grafts) in the patient's heart to bypass the narrowed or blocked coronary arteries. The aim is to ensure adequate blood flow to supply the heart with the required amount, thereby restoring its function [5].

The internal thoracic artery is predominant for the long-term patency of coronary artery grafts [6]. Factors influencing long-term patency include intraoperative graft torsion and graft flow restriction. Intraoperative evaluation is important to detect graft failure at an early stage. Recently, TTFM and IFI have been used [7].

TTFM is a simple, reliable, and reproducible method for intraoperative blood flow analysis, and many institutions have adopted this technique in recent years. On the other hand, contrast-enhanced IFI allows direct visual evaluation of graft flow, but the data obtained are qualitative, whereas quantitative data cannot be obtained [8].

In this case, the graft was evaluated using TTFM after anastomosis in the initial surgery, and the flow parameter of the left internal thoracic artery to the left anterior descending artery (LITA-LAD) was rather low at 9 mL/h, but other parameters such as pulsatility index (PI), diastolic filling index (PI), and diastolic filling (DF) were acceptable; however, we did not consider another modality evaluation of the graft because of the narrow perfusion zone of the LAD vessels in addition to the high degree of calcification. Surgery was completed, and subsequent graft angiography revealed a left internal thoracic artery dissection. Therefore, we decided to resect the dissection site. IFI was used to identify the dissection site, taking advantage of real-time evaluation in the operative field. Postoperative graft angiography revealed that the dissected site of LITA was resected without complications.

A retrospective study suggested that IFI could be used to detect dissection during initial surgery. However, considering the small number of vascular beds, the IFI was considered reasonable. In the future, we will consider performing the IFI in addition to the TTFM during each examination.

## Conclusions

We encountered a case of LITA dissection during coronary artery bypass surgery. During the initial operation, graft function was evaluated using TTFM; however, dissection was not detected. Subsequent postoperative imaging revealed a partial LITA dissection. During reoperation, the dissected portion of the LITA was excluded.

During reoperation, IFI allowed real-time visualization and identification of the dissection site. This case suggests the usefulness of IFI and is reported here.
